# A comparative study of the risk of delayed cerebral ischemia after clipping and coiling of ruptured intracranial aneurysms: a systematic review and meta-analysis

**DOI:** 10.3389/fneur.2026.1767989

**Published:** 2026-04-30

**Authors:** CaiYong Jiang, Jie Peng, Tao Luo, MingSheng Huang, Ai Chen, JianChao Xu, Wei Xiong, ZhiHui Tan, Jun Su

**Affiliations:** 1 Department of Neurosurgery, The People's Hospital of Nanchuan, Chongqing, China; 2 Department of Neurosurgery, The People's Hospital of Jianyang City, Chengdu, Sichuan Province, China

**Keywords:** clipping, coiling, delayed cerebral ischemia, meta-analysis, ruptured intracranial aneurysms

## Abstract

**Introduction:**

Delayed cerebral ischemia (DCI) is one of the main causes of disability and death following surgical treatment of ruptured intracranial aneurysms. Many studies combine data from patients undergoing clipping and coiling; however, this approach may be inappropriate. The purpose of this study was to compare the incidence of DCI between the two surgical methods and to explore, separately for each method, the predictive factors of DCI.

**Methods:**

This study systematically searched relevant articles on DCI after intracranial aneurysm surgery published in the PubMed, EMBASE, and Web of Science databases since their inception up to October 2025. After screening for inclusion criteria, RevMan software was used to perform a meta-analysis of the eligible studies.

**Results:**

This study included a total of 21 articles, including 5,358 patients. The results showed that there was a significant difference in the incidence of DCI after clipping and coiling. The combined OR value was 1.57 (95 % CI: 1.31–1.89), and the heterogeneity was within the acceptable range. The difference was statistically significant. In patients undergoing clipping, Fisher grade (OR 2. 11; 95 % CI: 1.57–2.84) and WFNS classification (OR 1.73; 95 % CI: 1.19–2.5) was a significant predictor of DCI, and the heterogeneity was low. Gender (OR 0.98; 95 % CI: 0.53–1.80), age (OR 1.34; 95 % CI: 0.13–2.55), hypertension (OR 0.97; 95 % CI: 0.75–1.25), diabetes (OR 1.48; 95 % CI: 0.77–2.83) and smoking history (OR 0.78; 95 % CI: 0.54–1.11) showed no statistical significance. In patients with interventional embolization, age (OR 3.34; 95 % CI: 1.27–5.41) and Fisher grade (OR 3.86; 95 % CI: 2.04–7.31) had a significant predictive effect on the occurrence of DCI.

**Conclusion:**

This study suggests that patients who have undergone clipping have a higher risk of DCI compared with patients undergoing coiling. Certain risk factors have shown a stronger predictive value in patients undergoing coiling. These conclusions require further validation by additional high quality studies.

## Introduction

Intracranial aneurysms are diseases caused by localized abnormal dilation of the arterial wall, which are common in the adult population globally. The incidence of intracranial aneurysms is 3%−5%. In addition, intracranial aneurysm rupture is the main cause of subarachnoid hemorrhage, which has a global incidence of (4–22)/100,000 (1, 2). For patients who survive subarachnoid hemorrhage, it is usually necessary to treat aneurysms by surgical clipping or coiling to control bleeding. However, many patients experience significant clinical deterioration after surgery, eventually leading to death or severe long-term dysfunction. One of the key reasons for this deterioration is delayed cerebral ischemia (DCI). DCI usually refers to cerebral ischemia occurring after the acute rupture of the intracranial aneurysm, which is common in the days to weeks following hemorrhage. The occurrence of DCI may be related to the reduction of cerebral blood flow caused by local hematomas or cerebral vasospasm. At present, there is still a lack of treatment methods that can effectively prevent such sequelae and improve the prognosis of patients (3–5).

Traditionally, surgical clipping has been regarded as the standard and effective method for the treatment of aneurysms. With the development of medical technology, coiling, which causes less trauma, has gradually become a popular treatment option. However, there are still few comparative studies on the occurrence of DCI after aneurysmal subarachnoid hemorrhage between these two methods. In addition, when analyzing the risk factors of DCI, many studies often discuss patients treated with clipping and coiling without distinguishing between the two surgical methods. This approach is not appropriate because there are significant differences in the mechanisms and pathophysiological effects of the two procedures. The mechanisms of DCI differ between the methods; the incidence may also vary, and combined analyses may obscure their independent risk characteristics (6–9). In this study, through systematic review and meta-analysis, the data from relevant controlled clinical studies were integrated to compare the differences in the occurrence of DCI after clipping and coiling. Unlike previous meta-analyses that combined the two surgical methods, this study not only explored the predictors of DCI for each surgical method separately but also included more recent studies, thereby enhancing the accuracy and reliability of the results.

## Methods

This study was conducted in strict accordance with the system review and meta-analysis of priority reporting items (PRISMA) statement specification.

### Literature search

We systematically searched the relevant literature in PubMed, EMBASE and Web of Science databases. The keywords used in the retrieval process include: “Intracranial Aneurysm” OR “Intracranial Aneurysms” OR “Cerebral Aneurysm” OR “Cerebral Aneurysms” OR “Brain Aneurysm” OR “Brain Aneurysms” OR “Anterior Cerebral Artery Aneurysm” OR “Anterior Cerebral Artery Aneurysms” OR “Anterior Communicating Artery Aneurysm” OR “Anterior Communicating Artery Aneurysms” OR “Basilar Artery Aneurysm” OR “Basilar Artery Aneurysms” OR “Middle Cerebral Artery Aneurysm” OR “Middle Cerebral Artery Aneurysms” OR “Posterior Cerebral Artery Aneurysm” OR “Posterior Cerebral Artery Aneurysms” OR “Berry Aneurysm” OR “Berry Aneurysms” OR “Posterior Communicating Artery Aneurysm” OR “Posterior Communicating Artery Aneurysms” OR “Giant Intracranial Aneurysm” OR “Giant Intracranial Aneurysms” OR “Intracranial Mycotic Aneurysm” OR “Intracranial Mycotic Aneurysms”? “Brain Ischemia” OR “Brain Ischemias” OR “Cerebral Ischemia” OR “Cerebral Ischemias” OR “Ischemic Encephalopathies” OR “Ischemic Encephalopathy”. The retrieval time range is from the establishment of the database to October 2025.

### Inclusion and exclusion criteria

We screened articles according to the following inclusion criteria: (1) patients with aneurysmal subarachnoid hemorrhage who developed DCI after clipping and coiling; (2) studies containing original data related to DCI. The exclusion criteria were as follows: (1) duplicate articles; (2) commentary articles, conference reports, and case reports.

### Data extraction

The preliminary screening articles were independently reviewed by two researchers (Jiang and Peng), with data extracted including author, nationality, study population, gender, average age, and surgical method. Any differences in the screening results were discussed and resolved by consensus between the two researchers.

### Assessment of risk of bias and quality

Two researchers (Jiang and Peng) used the Newcastle-Ottawa Scale (NOS) to independently evaluate the quality of the included studies. The scale consisted of nine items. Four items assessed the selection quality of the study, two items evaluated the comparability of groups, and the remaining three items assessed outcome quality and follow-up completeness. Based on the total scores on the scale, study quality was categorized into three levels: low quality ( ≤ 6 points), medium quality (7 points), and high quality (8–9 points). In cases where differences arose during the evaluation process, the two researchers reached an agreement through discussion and consultation.

### Statistical analysis

All statistical analyses were performed using Review Manager 5.4 software. If the heterogeneity test result (I^2^) was less than 50%, the fixed effect model was used for meta-analysis; otherwise, the random effect model was selected. For results exhibiting high heterogeneity, we further verified their stability through sensitivity analysis. Additionally, we conducted a leave-one-out analysis to assess the impact of individual studies on the pooled effect. A *p*-value less than 0.05 was considered statistically significant.

## Results

**Figure 1:**
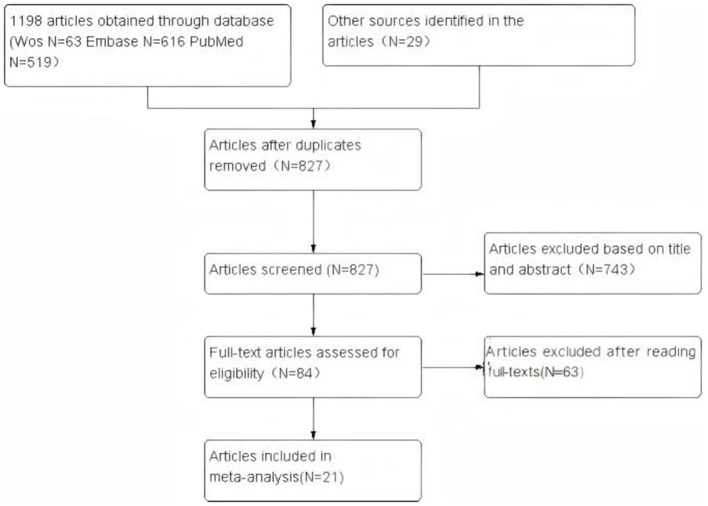
Flowchart of the article search performed.

The article screening process and results are shown in Figure 1. A total of 1,227 articles were obtained by searching PubMed, EMBASE, Web of Science, and other sources. After removing duplicate articles, 827 articles remained. Finally, 21 eligible studies were included, comprising 17 retrospective studies and four prospective studies. The basic characteristics of each study, such as author, nationality, study population, gender, and surgical method, are shown in Table 1. The scores of all included studies on the Newcastle-Ottawa Scale (NOS) ranged from 7 to 9 points, indicating high quality (Table 2, Table 3).

**Table 1:** Summary of characteristics of the included studies.

Study	Country	Participants	Gender	Mean Age (years)	Clipping	Coiling
Yasuhiro Kawabata 2010	Japan	102	M 40;W 62	None	77	25
Karol P. Budohoski (11)	UK	98	M 29;W 69	DCI 56;Non-DCI 57	67	34
Bradley A. Gross (12)	USA	255	M 63;W 192	None	203	52
Johannes Platz (14)	Germany	482	None	None	168	314
Jeremy J. Heit (17)	USA	16	M 5;W 11	56	6	10
Yung Ki Park (18)	Korea	412	None	DCI 56.1;Non-DCI 55.8	293	119
Liuwei Chen (19)	China	197	M 83;W 114	DCI 61.0;Non-DCI 59.0	42	155
Andrew M Nguyen (21)	USA	46	None	None	15	31
Yi-Bin Zhang (21)	China	730	M 278;W 452	DCI 55.6;Non-DCI 55.7	474	256
Ping Hu (22)	China	412	M 162;W 250	57	227	185
Tomofumi Takenaka (23)	Japan	128	M 41;W 87	Clipping 66.5;Coiling 58.0	106	22
Mengyuan Xu (24)	China	101	M 47;W 54	DCI 55.4;Non-DCI 59.2	48	53
Pei-Sen Yao (25)	China	360	M 190;W 170	DCI 56.2;Non-DCI 54.7	360	0
Lukas Goertz (26)	Germany	157	M 46;W 111	DCI 58.2;Non-DCI 53.9	157	0
Jun Su (28)	China	419	M 137;W 282	DCI 53.2;Non-DCI 52.6	419	0
Jie Wang (27)	China	534	M 225;W 309	54.7	534	0
Kehua Chen (30)	China	424	M 171;W 253	DCI 61.0;Non-DCI 58.0	0	424
Paul M. Foreman (13)	USA	150	None	None	79	71
Guojing Liu (15)	China	81	M 26;W 55	DCI 56.3;Non-DCI 49.5	66	15
Wanying Duan (16)	China	161	None	None	63	98
Mark Schembri (29)	Netherlands	90	M 27;W 63	DCI 59.1;Non-DCI 54.3	0	90

**Table 2:** Results of quality assessment using the NOS for the case–control study.

References	Selection	Comparability	Outcome	Total
Yasuhiro Kawabata (10)	^***^	^*^	^***^	7
Karol P. (11) 2012	^****^	^*^	^***^	8
Bradley A. Gross (12)	^****^	^*^	^***^	8
Johannes Platz (14)	^****^	^*^	^***^	8
Jeremy J. Heit (17)	^****^	^*^	^***^	8
Yung Ki Park (18)	^***^	^*^	^***^	7
Liuwei Chen (19)	^****^	^*^	^***^	8
Andrew M Nguyen (20)	^****^	^*^	^***^	8
Yi-Bin Zhang (21)	^****^	^*^	^***^	8
Ping Hu (22)	^****^	^*^	^***^	8
Tomofumi Takenaka (23)	^****^	^**^	^***^	9
Mengyuan Xu (24)	^****^	^*^	^**^	7
Pei-Sen Yao (25)	^***^	^*^	^***^	7
Lukas Goertz (26)	^****^	^*^	^***^	8
Jun Su (28)	^****^	^*^	^***^	8
Jie Wang (27)	^****^	^**^	^***^	9
Kehua Chen (30)	^***^	^*^	^***^	7

**Table 3:** Results of quality assessment using the NOS for the cohort studies.

References	Selection	Comparability	Outcome	Total
Paul M. Foreman (13)	^****^	^**^	^**^	8
Guojing Liu (15)	^****^	^*^	^***^	8
Wanying Duan (16)	^****^	^*^	^**^	7
Mark Schembri (29)	^***^	^*^	^***^	7

### Data analysis

**Figure 2:**
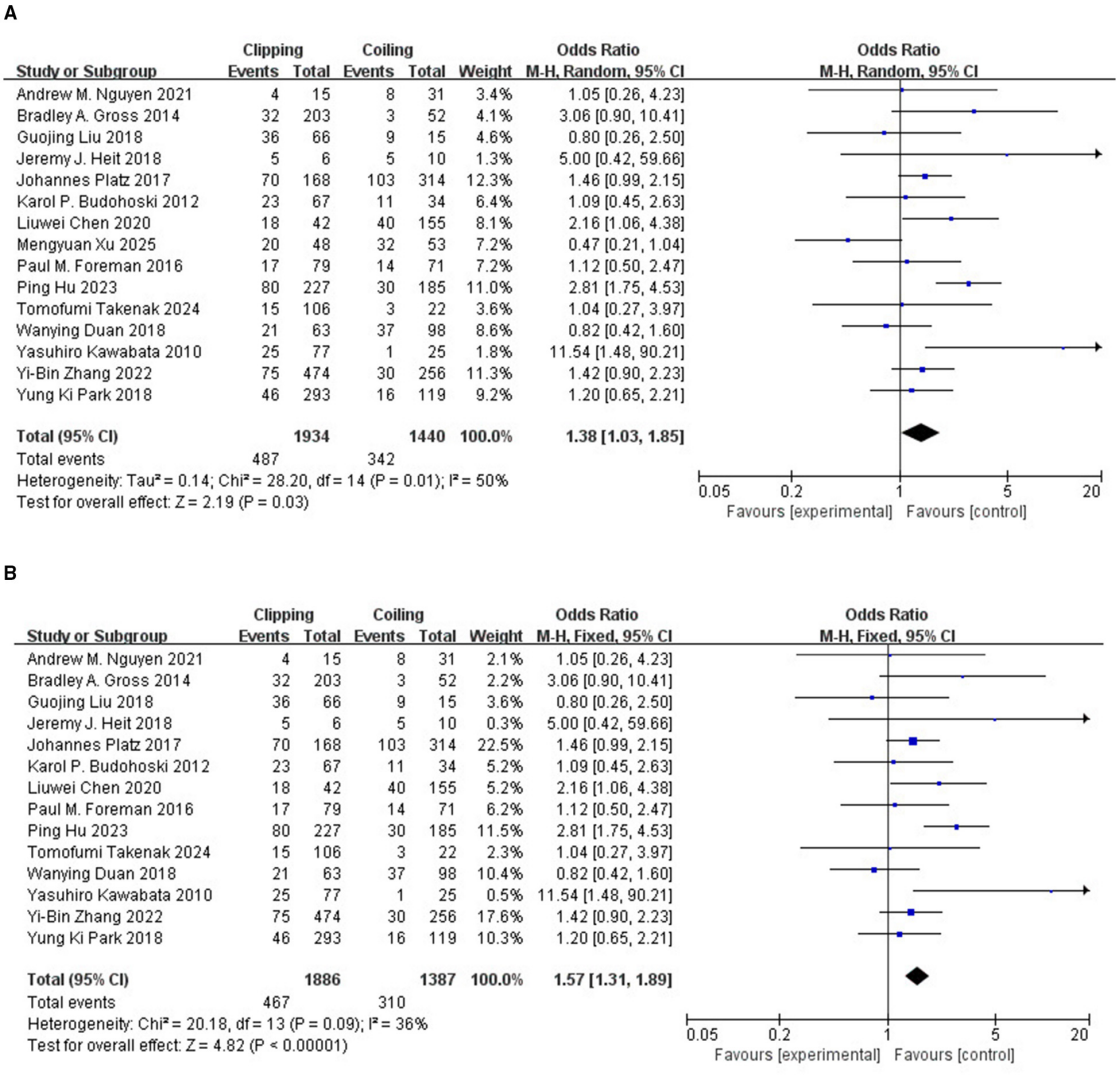
**(A)** Forest plot of DCI risk comparison between clipping and coiling. **(B)** Forest plot of DCI risk comparison between adjusted clipping and coiling.

To evaluate the difference in the incidence of DCI after clipping and coiling, a total of 15 studies involving 3,374 patients were included in the analysis (10–24). The preliminary analysis results (Figure 2A) showed that the combined OR for the risk of DCI between the two groups was 1.38 (95% CI: 1.03–1.85), with high heterogeneity (*I*^2^ = 50%). After adjusting for heterogeneity through the leave-one-out analysis method, the results are shown in Figure 2B, with an OR of 1.57 (95% CI: 1.31–1.89). The heterogeneity was reduced to an acceptable level (*I*^2^ = 36%), and the difference was statistically significant (P <0.05), indicating a higher incidence of DCI after clipping compared to coiling. After sensitivity analysis, it was found that Xu M's research excluded patients with local skull defects caused by surgery, which led to underestimation of the effect. However, the adjusted study was closer to the true correlation strength.

### Clipping group

A total of 4 articles were included to analyze the risk factors of DCI after clipping, involving 1,470 patients (25–28). Among them, four studies assessed the effects of age, gender, and Fisher grade, three studies assessed hypertension, diabetes, and smoking history, and two studies assessed WFNS (World Federation of Neurosurgical Societies) grade.

**Figure 3:**
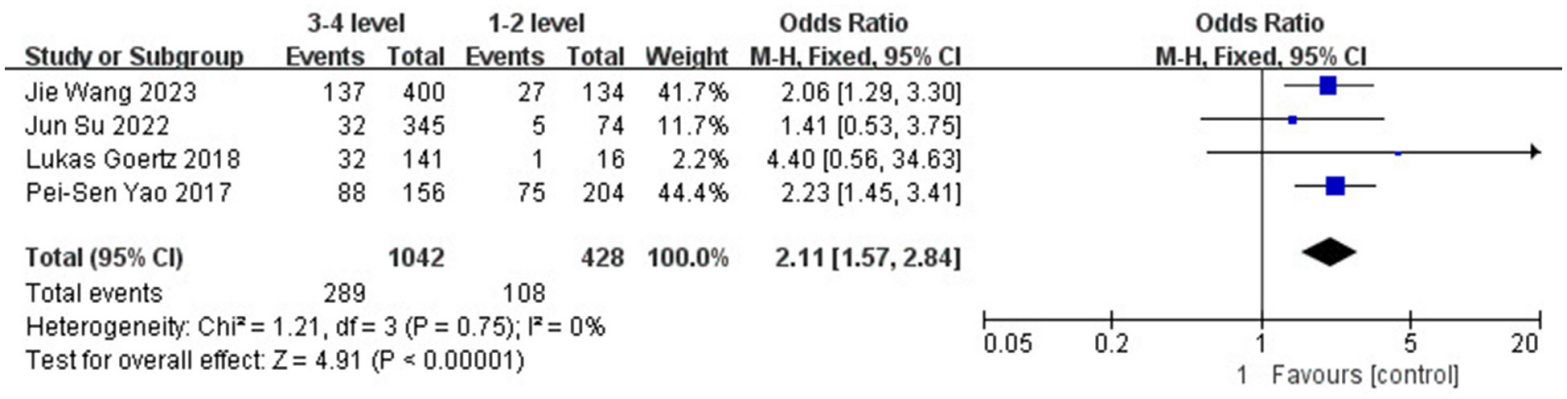
Forest plot of the correlation between Fisher grade and DCI incidence after clipping.

**Figure 4:**
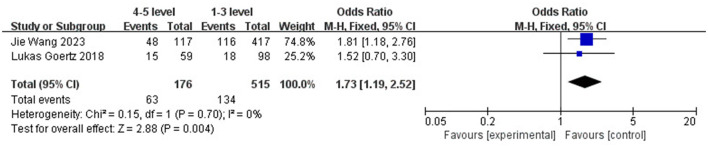
The forest plot of the correlation between WFNS grade and DCI incidence after clipping.

The results showed that Fisher grade (OR 2.11; 95% CI: 1.57–2.84; *I*^2^ = 0%; Figure 3) and WFNS grade (OR 1.73; 95% CI: 1.19–2.52; *I*^2^ = 0%; Figure 4) have a significant predictive effect on the occurrence of DCI after clipping (*P* < 0.05), and the heterogeneity is low.

**Figure 5:**
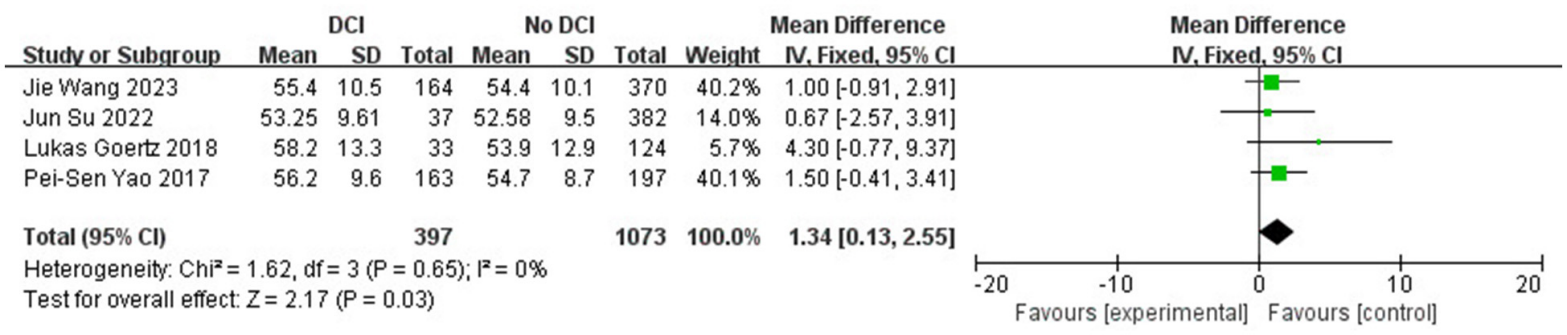
Forest plot of correlation between age after clipping and incidence of DCI.

**Figure 6:**
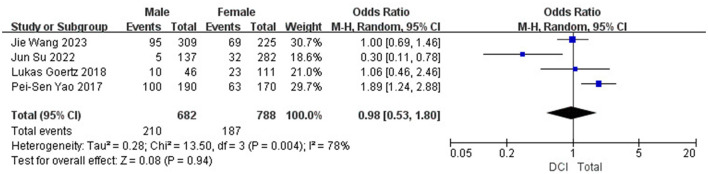
Forest plot of the correlation between gender and DCI incidence after clipping.

**Figure 7:**
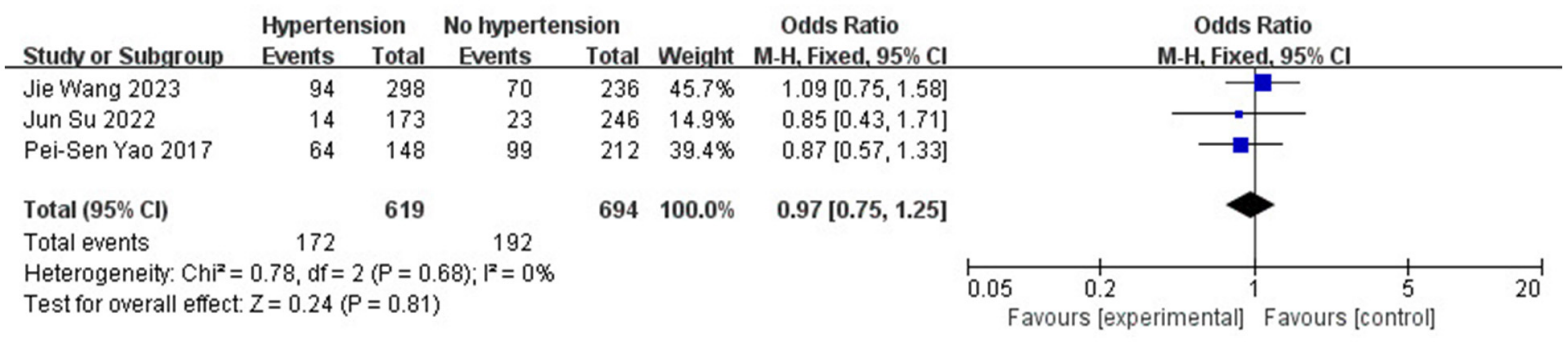
Forest plot of the correlation between hypertension and DCI incidence after clipping.

**Figure 8:**
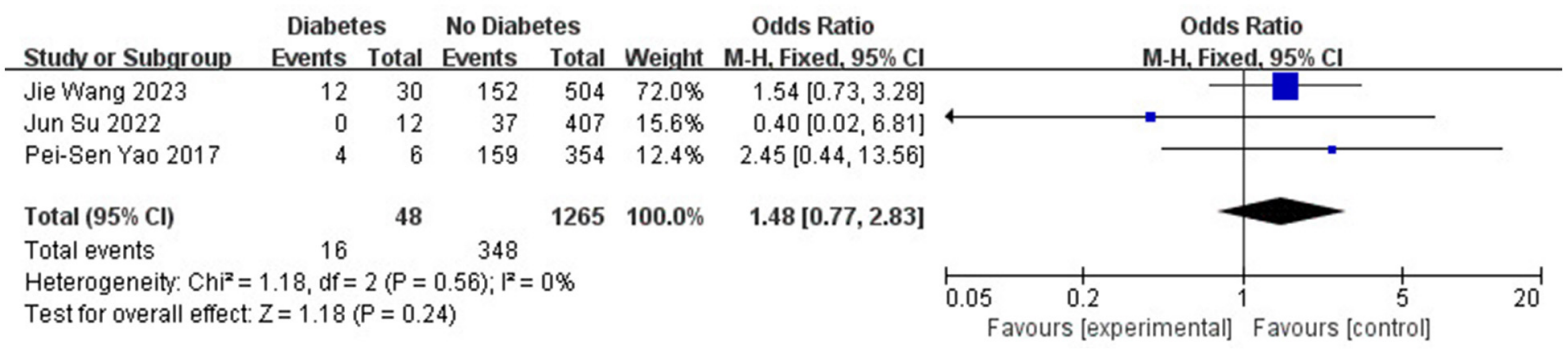
Forest plot of the correlation between diabetes and DCI after clipping.

**Figure 9:**
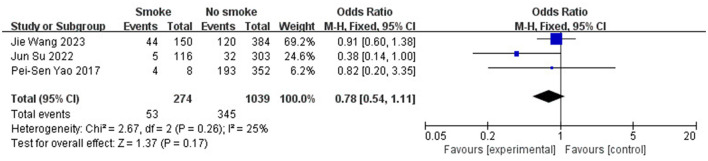
Forest plot of the correlation between smoking and DCI incidence after clipping.

Age (OR 1.34; 95% CI: 0.13–2.55; *I*^2^^2^ = 0%; Figure 5), gender (OR 0.98; 95% CI: 0.53–1.80; *I*^2^ = 78%; Figure 6), hypertension (OR 0.97; 95% CI: 0.75–1.25; *I*^2^ = 0%; Figure 7), diabetes (OR 1.48; 95% CI: 0.77–2.83; *I*^2^^2^ = 0%; Figure 8), and smoking history (OR 0.78; 95% CI: 0.54–1.11; *I*^2^^2^ = 25%; Figure 9) were not statistically significant (*P* > 0.05), suggesting that they have no significant predictive value for the occurrence of DCI after clipping.

### Coiling group

**Figure 10:**
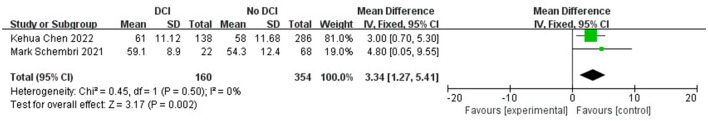
Forest plot of the correlation between age and DCI incidence after coiling.

**Figure 11:**
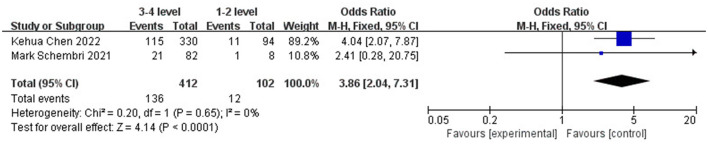
Forest plot of the correlation between Fisher grade and DCI incidence after coiling.

**Figure 12:**
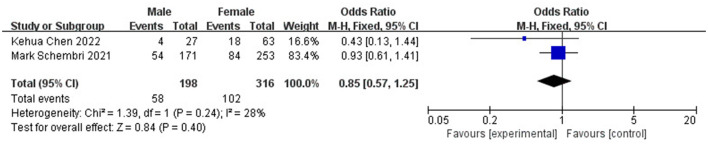
Forest plot of the correlation between gender and DCI incidence after coiling.

To evaluate the risk factors of DCI after coiling, a total of two studies were included for analysis, covering 514 patients (29, 30). The results showed that age (OR 3.34, 95% CI: 1.27–5.41, *I*^2^ = 0%; Figure 10) and Fisher's classification (OR 3.86, 95% CI: 2.04–7.31, *I*^2^ = 0%; Figure 11) have significant predictive value for the occurrence of DCI after coiling (P <0.05). In contrast, gender (OR 0.85, 95% CI: 0.57–1.25, *I*^2^ = 28%; Figure 12) was not statistically significant (*P* > 0.05).

## Discussion

At present, there are few studies that independently discuss and compare DCI after clipping and coiling, and combined analyses may introduce errors due to the different mechanisms and incidences of the two methods. In this study, a meta-analysis found a significant difference in the incidence of DCI between the two surgical methods, and the associated risk factors were systematically reviewed. The results showed that Fisher Grade was predictive of DCI occurrence following both surgical methods. Additionally, WFNS grade has predictive value for DCI after clipping and age has predictive value for DCI after coiling. Notably, the predictive value of Fisher Grade after coiling is more significant compared to clipping.

A meta-analysis conducted by Xiao ZK et al. showed that gender, Fisher grade, WFNS grade, hypertension, and white blood cell (WBC) count were independent risk factors for DCI. However, in this study, hypertension was not a predictor of DCI after clipping, and gender did not show predictive significance after either procedure. This discrepancy may be related to the choice of subjects: Xiao ZK's study combined patients who underwent clipping and coiling, whereas this study analyzed the two procedures separately, thereby revealing subgroup-specific prognostic patterns. Furthermore, several previous meta-analyses yielded results inconsistent with our findings. A meta-analysis by Shen Z et al. showed no significant difference in the incidence of DCI between clipping and coiling in patients with unruptured aneurysms. Similarly, Chai CL and Xiao ZK reported no significant difference in DCI risk between the two surgical methods in patients with ruptured aneurysms. In contrast, this study observed that clipping and coiling exhibit distinct patterns of DCI occurrence (31–33). This inconsistency may be attributed to high heterogeneity in previous meta-analyses, characterized by variations in patient baseline characteristics, aneurysm rupture status, perioperative management, and DCI definitions. Furthermore, previous meta-analyses have prioritized clinical outcomes over mechanism-based differences, which may obscure the specific pathophysiological effects of different surgical procedures on DCI.

There are significant differences in the mechanisms between clipping and coiling. The occurrence of DCI after clipping may be related to intraoperative aneurysm rupture, excessive clipping, and other factors. Intraoperative aneurysm rupture will prolong the operation time, increase the duration of parent artery occlusion, and aggravate peripheral vascular injury. Improper clipping position or excessive clipping can lead to an increase in subarachnoid hemorrhage. Postoperative blood cell disintegration releases more vasospastic substances. In addition, excessive clipping may also cause vascular endothelial injury, induce vasospasm, and even thrombosis, ultimately promoting the occurrence of DCI. Clipping was associated with cerebral retraction and direct vascular manipulation. However, the mechanisms of DCI after coiling are related to the process of aneurysm embolization, coil displacement, and microthrombosis. Coiling avoids craniotomy and brain retraction; however, it is associated with endothelial damage, microthrombosis, and subtle hemodynamic shifts within the aneurysm and distal vasculature (9, 29, 30, 34–36). Boulouis et al. proposed that mechanical stimulation during interventional procedures can lead to abnormal contraction of cerebral arteries, affect blood perfusion in the distal blood supply area, and then cause cerebral ischemia and hypoxia, resulting in neurological dysfunction or irreversible cerebral infarction (37).

Studies have shown that inflammatory factors can stimulate vascular endothelial cells, induce an immune inflammatory response of the vascular wall, and promote smooth muscle contraction. In addition, inflammatory factors can promote the up-regulation of endothelin-1 synthesis, further exacerbating vasoconstriction and thereby increasing the risk of DCI. Surgical trauma is an important cause of the release of inflammatory factors. To fully expose the parent artery and the neck of the aneurysm, clipping often requires a certain degree of vascular traction and dissection. Consequently, the surgical trauma is significant. In contrast, coiling is less traumatic and usually results in lower levels of postoperative inflammatory factors. This may be one of the reasons for the difference in the incidence of DCI between the two procedures (38, 39).

Age is an effective predictor of DCI, and its mechanism may be related to the poor baseline condition of the vascular wall in elderly patients, such as thinning of the elastic layer of the intima, and a tendency to develop atheromatous plaques or mural thrombi. During the operation, these unstable plaques or thrombi may dislodge, forming microemboli that block distal arterioles and lead to cerebral ischemia in the corresponding blood supply area (40).

Studies have pointed out that smoking is a risk factor for intracranial aneurysm formation, growth of primary aneurysms, and aneurysm rupture. Tan J et al. further confirmed that smoking is an independent risk factor for *de novo* intracranial aneurysms and established a corresponding risk prediction model; this model has been verified by multi-center external validation (41–43). These findings highlight the significant impact of smoking on aneurysm development. However, the effect of smoking on DCI remains unclear and warrants further investigation. Cigarette smoke contains complex constituents, some of which may cause cerebral vasoconstriction, while other components may have vasodilatory effects. Studies have suggested that smoking may promote the release of nitric oxide by activating central acetylcholine neurons, thereby causing cerebral vasodilation (44). Conversely, Uchida S et al. believed that smoking may affect vascular function by damaging vascular endothelium and elevating protein kinase C activity (45). Therefore, the specific mechanisms and effects of smoking on DCI remain unclear and warrant further study.

Fisher grade, WFNS grade and Hunt-Hess grade are common tools for evaluating the condition of patients with subarachnoid hemorrhage (aSAH). The analysis of this study showed that patients with the higher Fisher grade and WFNS grade had a significantly increased risk of DCI. Several studies have shown that higher scores are associated with greater intracranial hemorrhage, higher intracranial pressure, and more pronounced cerebral hypoperfusion. These factors collectively lead to an increased risk of DCI and are closely related to poor prognosis in patients (25–30, 33). However, due to the limited number of studies examining the Hunt-Hess grade in relation to the two surgical methods, this analysis was unable to evaluate the effect of the Hunt-Hess grade independently.

There are some limitations in the meta-analysis conducted in this study. First, the number of independent studies on clipping and coiling is limited, resulting in only two studies included in the embolization group. It is difficult to analyze potential risk factors such as smoking, diabetes, and hypertension, which may have predictive value for the occurrence of DCI after coiling. Due to insufficient articles, it is also impossible to analyze the predictive effect of Hunt Hess grading and hematological indicators on DCI in the coiling group. Second, all included studies employ observational designs, and observer bias is inevitable.

## Conclusion

Current studies suggest that, compared with coiling, the incidence of DCI after clipping may be higher. Additionally, the predictive effect of some risk factors in coiling appears to be more significant. Therefore, it is suggested that the two surgical methods should be studied separately to explore the risk factors of DCI in future research; this approach may be expected to improve the accuracy and validity of the analysis. However, high-quality studies comparing the two procedures remain limited, and additional research is needed to verify this conclusion.

## Data Availability

The original contributions presented in the study are included in the article/supplementary material, further inquiries can be directed to the corresponding author.
